# Searching fundamental information in ordinary differential equations. Nondimensionalization technique

**DOI:** 10.1371/journal.pone.0185477

**Published:** 2017-10-03

**Authors:** J. F. Sánchez Pérez, M. Conesa, I. Alhama, F. Alhama, M. Cánovas

**Affiliations:** 1 Network Simulation Research Group, Universidad Politécnica de Cartagena, Cartagena, Spain; 2 Metallurgical and Mining Engineering Department, Universidad Católica del Norte, Antofagasta, Chile; China University of Mining and Technology, CHINA

## Abstract

Classical dimensional analysis and nondimensionalization are assumed to be two similar approaches in the search for dimensionless groups. Both techniques, simplify the study of many problems. The first approach does not need to know the mathematical model, being sufficient a deep understanding of the physical phenomenon involved, while the second one begins with the governing equations and reduces them to their dimensionless form by simple mathematical manipulations. In this work, a formal protocol is proposed for applying the nondimensionalization process to ordinary differential equations, linear or not, leading to dimensionless normalized equations from which the resulting dimensionless groups have two inherent properties: In one hand, they are physically interpreted as balances between counteracting quantities in the problem, and on the other hand, they are of the order of magnitude unity. The solutions provided by nondimensionalization are more precise in every case than those from dimensional analysis, as it is illustrated by the applications studied in this work.

## Introduction

The search of the dimensionless groups of a physical or engineering problem is a basic subject which has interest to most of the researchers, since any system of equations, containing mathematical formulation of physical laws can be represented as a relation between dimensional quantities [1,2]. To attain this aim, two techniques are currently used: dimensional analysis and nondimensionalization. The first one is a classical approach and, in spite of its detractors [3,4], there are many publications and books about this topic [5–10]. According to Gibbings [11], the delight of dimensional analysis is the combination of its great utility with a demanding intellectual rigor. However, it often has imperfections as a result of a careless treatment, and it is difficult to offer a defense against the assertion about that this technique is only effective because the correct answer is previously known. Dimensional analysis is a formidable tool in the most difficult problems, but their results are sometimes incomplete, so it requires explanations in order to get a complete understanding of the solutions. Nevertheless, it is rare to find applications of dimensional analysis to linear and nonlinear problems, ruled by ordinary differential equations and coupled systems of these equations. Problems such as the motion of a simple pendulum, the flow of a heavy fluid through a spillway, fluid motion in pipes, the motion of a body in a fluid and other problems, which are studied as typical examples in books about dimensional analysis [11, 12, 13], in order to demonstrate that this technique is worth applying. Even though they are more difficult to analyze, applications to coupled partial differential equations, in their discriminate version, are frequently published in the scientific literature [14–16].

The technique of nondimensionalization dates back to a long time. In 1935, Ruark [17] wrote: ‘*Inspectional analysis* (the name he gives to nondimensionalization) *consists in transforming the equations of the problem*, *differential or otherwise*, *so that all the variables are dimensionless*. *Simple inspection then shows how these dimensionless variables are related… A formal solution of the equations may then be obtained by writing each dependent dimensionless variable as a power series in which the arguments are the independent dimensionless variables*.’ For some authors, in fact, nondimensionalization and dimensional analysis provide the same information [18], while for others the technique of nondimensionalization may reveal more accuracy than dimensional analysis and, in that sense, it is more powerful [19]. But, as occurs with dimensional analysis, nondimensionalization is applied to complex problems ruled by coupled partial differential equations [4, 20, 21], but it is scarcely found in problems ruled by ordinary differential equations. As we will see later, the reason of that lies in that there is more than one reference to make dimensionless the variables, and this technique is rarely applied to this kind of equation.

In this work, we introduce the nondimensionalization, in a formal protocol, whereby dimensionless variables are defined being their range of variation extends within the interval [0–1], covering it, either completely or near completely. The resulting equations are not only dimensionless, but also what it is called normalized, in the sense their solutions are universal. Suitable references, related between them, must be chosen to satisfy this assumption.

In this way, once the dimensionless governing equations are established, each of their addends is the product of two factors, as it is appreciated in the applications: one formed by the dimensionless variables and/or their changes (derivatives), and the other formed by a grouping of parameters of the problem. Assuming that the mean value of the first factor of all the addends is of the order of magnitude unity—an admissible hypothesis unless the nonlinearity of the problem is too acute, as we will check in every example—, the other factors must also be of the same order of magnitude. Therefore, the ratios of the last factors (the dimensionless numbers) have an order of magnitude unity, and may be physically interpreted as balances of the quantities counteracting in the process, within the temporal or geometrical domain of the problem.

The essential for an optimal nondimensionalization, what might be called normalized nondimensionalization, resides in a correct choice of the magnitudes of reference that convert the dependent and independent variables of the problem to their dimensionless form. These references can be explicitly contained in the statement of the problem or, on the contrary, be implicitly; In this case, they can be call hidden variables. In any case, it is necessary that they relate to each other. For example, if the time reference is the time interval between t = 0 y t = t_0_, then the reference for the dependent variable associated should be the difference between of the values of this one for those times, and not any other value, although having the same physical dimension.

If the selection of references is such that it confines the values of the variables in the interval [0–1], as has already been said above, the resulting equation, dimensionless and normalized, will allow determination of the dimensionless groups of the problem. These groups will be of order of magnitude unity and with clear physical meaning, in terms of the balance of the variables that interact in the problem.

The functional dependence between these dimensionless groups leads to the solution of the problem and the expression of the unknowns in terms of the other parameters.

The above protocol provides solutions that, in general, overcome the results of dimensional analysis, which never refers to any order of magnitude of the unknowns sought. Gibbings [11] is one of the few recent authors that: i) clearly ascribes a physical significance to some of the classical dimensionless numbers (Reynolds, Euler, Grashof…), while recognizing that this is not always possible, and ii) affirms that these numbers, neither give a direct numerical measure of the unknowns nor an order of magnitude of the ratios that they represent. The reason, clearly, is that the variables in the relevant list, when it is applied the dimensional analysis, are generally chosen without any kind of spatial and/or temporal connection between them.

Three applications, that include nonlinear problems, are presented to illustrate the proposed protocol for nondimensionalization: a pendulum over an accelerated platform, a pendulum over a sliding support and simple interaction between two species. In all of them, the solutions provided by nondimensionalization have more accuracy (it is exact in the second application) than those given by dimensional analysis. Discussions about the choice of suitable references (whether or not established in the statement of the problem) and their relations, the existence of hidden references, references for asymptotic problems and other aspects related to the process of nondimensionalization, are discussed in each application. Finally, the solutions are checked by numerically solving each problem using the network method [22,23].

## Application 1. Pendulum over an accelerated platform

**Fig 1:**
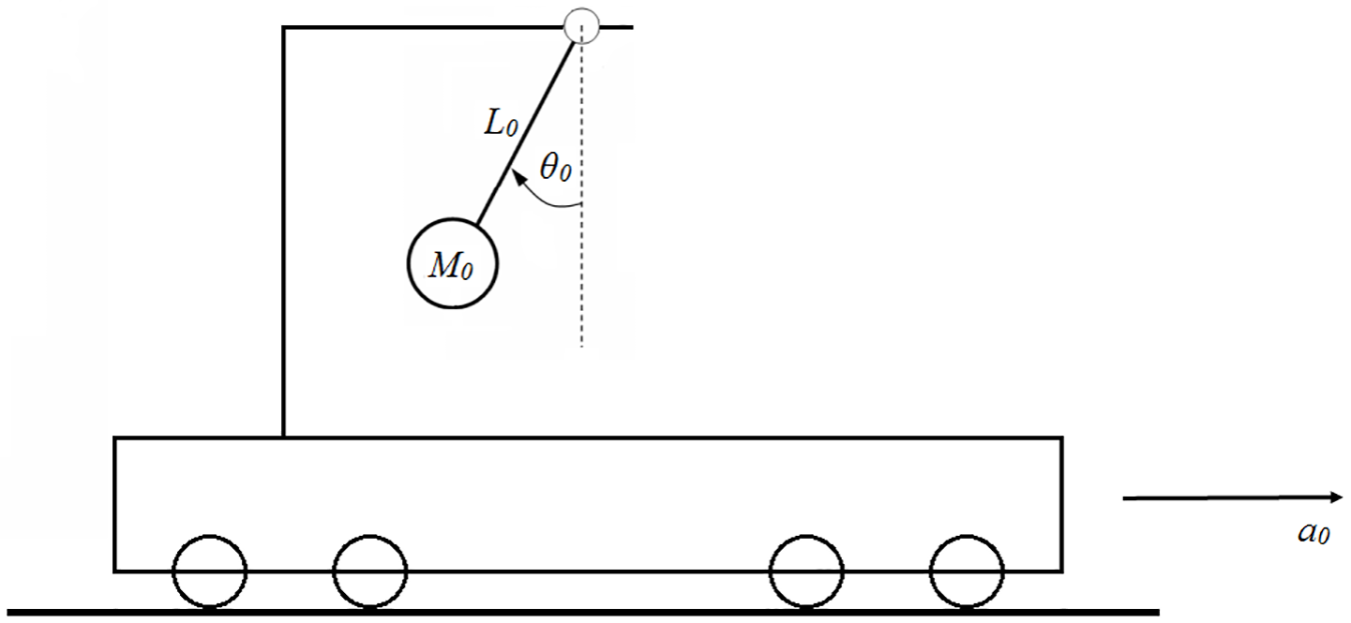
Physical scheme of the problem.

A platform with acceleration a_o_, transports a physical pendulum of length l_o_ and mass m_o_, as shown in Fig 1. The angle θ_o_, measured from the vertical, will be the displacement of the pendulum and, releasing it with zero velocity, give rise to oscillations of period T_o_. The aim is to study this period.

The platform-pendulum system has an equilibrium position defined by the angle θ_e_, around which the oscillations take place. This angle is related to a_o_ and g by the expression

$$\text{tg}\left(\theta_{\text{e}}\right)=\frac{{\text{a}}_{\text{o}}}{\text{g}}$$ (1)

**Fig 2:**
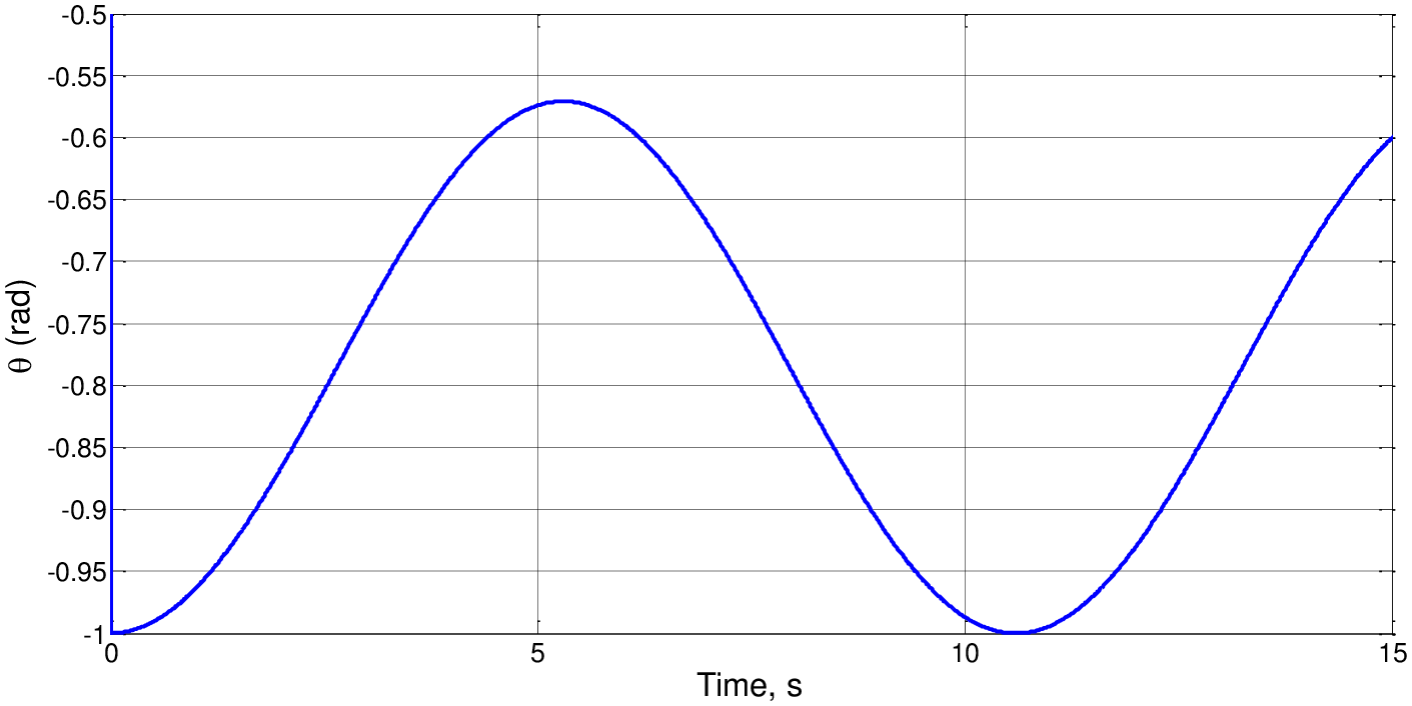
θ(t) for l_o_ = 4, a_o_ = g = 1, θ_o_ = -1 and *d*θ/*d*t_(t = 0)_ = 0.

For example, for l_o_ = 4 and a_o_ = g = 1, we obtain θ_e_ = π/4 (0.785) rad. Fig 2 shows the solution θ(t) for the initial conditions θ_o_ = -1 y *d*θ/*d*t_(t = 0)_ = 0. Note that θ_o_ is quite close to the average value of the maximum and minimum angular displacements, which are always negative.

Firstly, we will apply the nondimensionalization process to this problem. Without losing generality, we fit the study, initially, to cases with small values of θ_e_ and θ_o_, and large values of θ_o_ compared with θ_e_; i.e., θ_o_-θ_e_≈θ_o_.

Under this assumption, which implies cos(θ_o_)≈1 and sen(θ_o_)≈θ_o_, the governing equation is

$${\text{l}}_{\text{o}}\left(\frac{{\text{d}}^{2}\theta }{{\text{dt}}^{2}}\right)\;+\;{\text{a}}_{\text{o}}\;+\;\text{g}\theta_{\text{o}}=0$$ (2)

Choosing the references θ_o_ and t_o_ (the time necessary to reach the lowest point of the way from the initial position), the dimensionless variables are written as θ’ = θ/θ_o_ and t’ = t/t_o_. Substituting them in the Eq (1) gives the dimensionless form of the governing equation, which is

$${\text{l}}_{\text{o}}\left(\frac{\theta_{0}}{{\text{t}}_{0}^{2}}\right)\left(\frac{{\text{d}}^{2}\theta \prime }{{\text{dt}}^{\prime 2}}\right)\;+\;{\text{a}}_{\text{o}}\;+\;{\text{g}\theta }_{\text{o}}\theta \prime =0$$ (3)

This equation provides three coefficients:

$${\text{C}}_{1}={\text{l}}_{\text{o}}\left(\frac{\theta_{0}}{{\text{t}}_{0}^{2}}\right),\;\;\;\;\;\;\;\;{\text{C}}_{2}={\text{a}}_{\text{o}},\;\;\;\;\;\;\;\;{\text{C}}_{3}={\text{g}\theta }_{\text{o}}$$ (4)

and two dimensionless groups (by dividing the two first coefficients by the last one):

$$\pi_{1}=\left(\frac{{\text{l}}_{\text{o}}}{{\text{gt}}_{0}^{2}}\right),\;\;\;\;\;\;\;\;\pi_{2}=\left(\frac{{\text{a}}_{\text{o}}}{{\text{g}\theta }_{\text{o}}}\right)$$ (5)

Then, the order of magnitude of t_o_ is given by the expression

$${\text{t}}_{\text{o}}=\sqrt{\left(\frac{{\text{l}}_{\text{o}}}{\text{g}}\right)}\Psi \left(\frac{{\text{a}}_{\text{o}}}{{\text{g}\theta }_{\text{o}}}\right)$$ (6)

The application of dimensional analysis begins with the list of relevant variables of the problem. These are: l_o_, g, a_o_, θ_o_ and the unknown T_o_ (m_o_ is not relevant, since is a common proportionality factor of the two forces that counteract in the problem, weight and inertia). The choice of t_o_, rather than T_o_, is more appropriate since it is an unknown that is directly related to θ_o_. However, such a choice is never made by the dimensional analysis. The list provides three dimensionless groups

$$\pi_{1}=\left(\frac{{\text{l}}_{\text{o}}}{{\text{gT}}_{0}^{2}}\right),\;\;\;\;\;\;\;\;\pi_{2}=\left(\frac{{\text{a}}_{\text{o}}}{{\text{g}\theta }_{\text{o}}}\right),\;\;\;\;\;\;\;\;\pi_{3}=\theta_{\text{o}}$$ (7)

and a less precise solution for the unknown

$${\text{t}}_{\text{o}}=\sqrt{\left(\frac{{\text{l}}_{\text{o}}}{\text{g}}\right)}\Psi \left(\frac{{\text{a}}_{\text{o}}}{\text{g}},\;\theta_{\text{o}}\right)$$ (8)

The solution given by nondimensionalization for cases with large values of θ_o_ can be obtained using the series expansion of sin and cos functions:

$$\text{sen}\left(\text{k}\theta \right)=\text{k}\theta \;-\;\frac{{\left(\text{k}\theta \right)}^{3}}{3!}\;+\;\frac{{\left(\text{k}\theta \right)}^{5}}{5!}\;-\;\ldots$$

$$\text{cos}\left(\text{k}\theta \right)=1\;-\;\frac{{\left(\text{k}\theta \right)}^{2}}{2!}\;+\;\frac{{\left(\text{k}\theta \right)}^{4}}{4!}\;-\;\ldots .$$

Retaining the two first terms of the series (using more terms, it leads to the same results), the governing equations can be written in the form

$${\text{l}}_{\text{o}}\left(\frac{{\text{d}}^{2}\theta }{{\text{dt}}^{2}}\right)\;+\;{\text{a}}_{\text{o}}\;\left(1\;-\;\frac{{\left(\theta \right)}^{2}}{2!}\right)\;+\;\text{g}\;\left(\text{a}\theta -\;\frac{{\left(\theta \right)}^{3}}{3!}\right)=0$$ (9)

and its corresponding dimensionless form

$${\text{l}}_{\text{o}}\left(\frac{\theta_{0}}{{\text{t}}_{0}^{2}}\right)\left(\frac{{\text{d}}^{2}\theta \prime }{{\text{dt}}^{\prime 2}}\right)\;+\;{\text{a}}_{\text{o}}\;\left(1\;-\;\frac{{\left(\theta_{\text{o}}\right)}^{2}}{2!}\theta^{\prime 2}\right)\;+\;\text{g}\;\left(\theta_{\text{o}}\theta \prime \;-\;\frac{{\left(\theta_{\text{o}}\right)}^{3}}{3!}\theta^{\prime 2}\right)=0$$ (10)

The coefficients of this equation are

$${\text{C}}_{1}={\text{l}}_{\text{o}}\left(\frac{\theta_{0}}{{\text{t}}_{0}^{2}}\right),\;\;\;\;\;\;\;\;{\text{C}}_{2}={\text{a}}_{\text{o}},\;\;\;\;\;\;\;\;{\text{C}}_{3}={\text{g}\theta }_{\text{o}},\;\;\;\;\;\;\;\;{\text{C}}_{4}={\text{a}}_{\text{o}}\left(\frac{{\left(\theta_{\text{o}}\right)}^{2}}{2!}\right),\;\;\;\;\;\;\;\;{\text{C}}_{5}=\text{g}\left(\frac{{\left(\theta_{\text{o}}\right)}^{3}}{3!}\right)$$ (11)

and the independent dimensionless groups formed by these coefficients (dividing by the last and reorganizing them)

$$\pi_{1,\text{f}}=\left(\frac{{\text{l}}_{\text{o}}}{{\text{gt}}_{0}^{2}}\right),\;\;\;\;\;\;\;\;\pi_{2,\text{f}}=\left(\frac{{\text{a}}_{\text{o}}}{\text{g}}\right),\;\;\;\;\;\;\;\;\pi_{3,\text{f}}=\theta_{\text{o}}$$ (12)

So that, the solution

$${\text{t}}_{\text{o}}=\sqrt{\left(\frac{{\text{l}}_{\text{o}}}{\text{g}}\right)}\Psi \left(\frac{{\text{a}}_{\text{o}}}{\text{g}},\;\theta_{\text{o}}\right)$$ (13)

is the same as that provided by dimensional analysis, substituting t_o_ for the period T_o_.

To check the reliability of the results given by the nondimensionalization, the cases of Table 1 were run numerically (simulation is carried out by the network method). To simplify the study, *d*θ/*d*t_(t = 0)_ = 0.

**Table 1:** Cases for the first application.

Case	1	2	3	4	5	6	7	8	9	10	11	12	13
**l**_**o**_	1	2	4	1	1	1	1	2	4	1	3	1	3
**a**_**o**_	1	2	4	1	0.5	0.25	1	1	1	1	6	1	6
**g**	10	20	40	10	10	10	10	20	40	10	30	10	30
**θ**_**o**_	0.5	0.5	0.5	0.1	0.05	0.025	1	0.05	0.025	0.75	1.5	0.05	0.1

**Fig 3:**
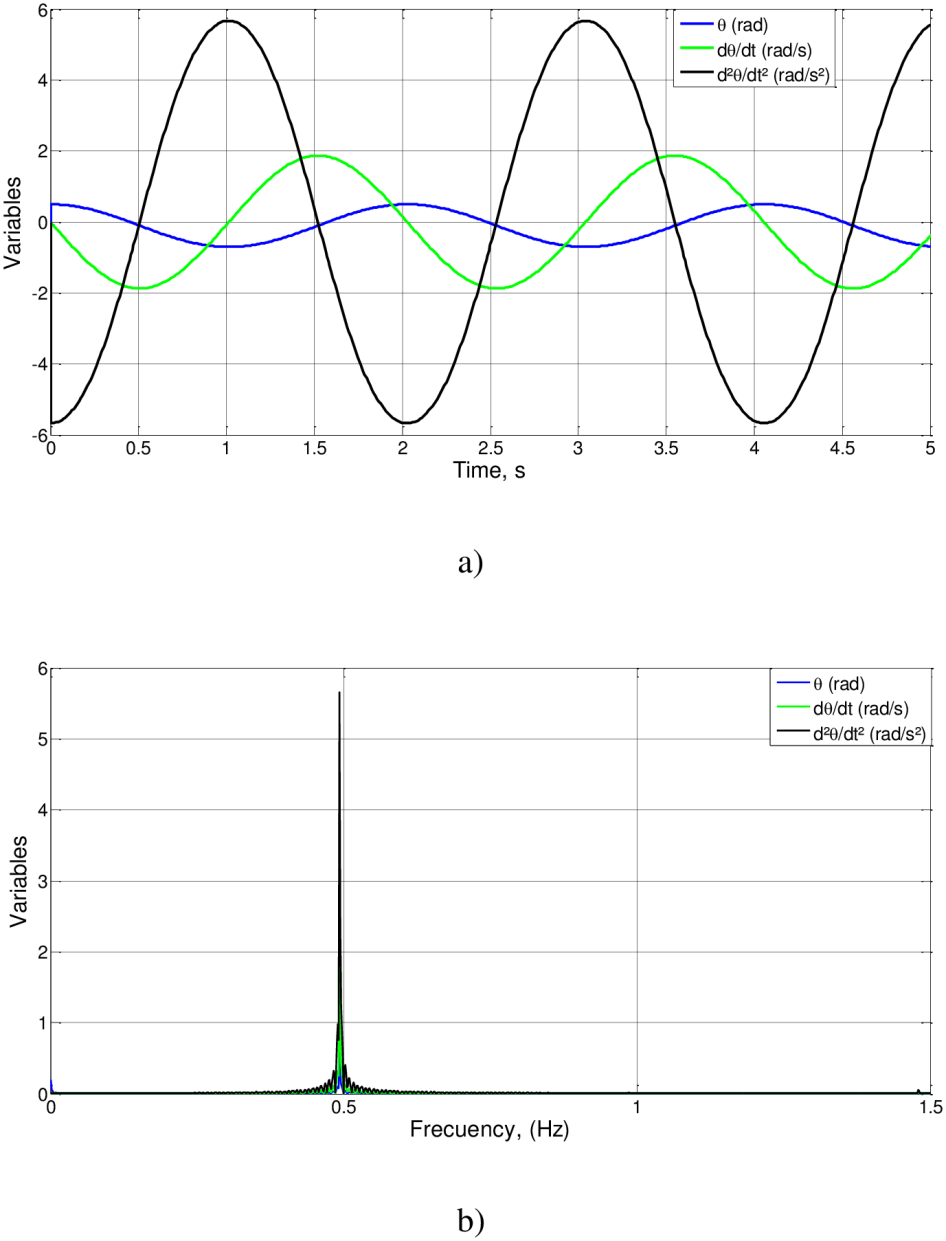
a): θ(t) and *d*θ/*d*t for cases 1 to 3, b): FFT spectra of cases 1 to 3.

We first check cases 1 to 3, related to proportional changes in the parameters l_o_, g y a_o_ and a large value for θ_o_. The results of the simulation (θ(t), *d*θ/*d*t and FFT spectra) are shown in Fig 3. For the three cases, t_o_ = 0.5069, T_o_ = 2.0275 and f_o_ = 0.4932. As (l_o_/g)^1/2^ = 0.3162, the value of the unknown function is Ψ(0.1,0.5) = 0.5069/0.3162 = 1.6030, of an order of magnitude unity, as expected.

**Fig 4:**
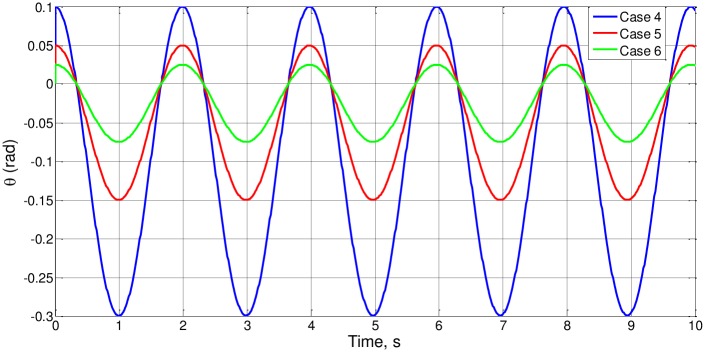
θ(t) for cases 4 to 6.

After, we check cases 4–6, studying proportional changes in the parameters θ_o_ and a_o_ for small angles of θ_o_. The solution θ(t) is shown in Fig 4, and t_o_ has the same value for the three cases, t_o_ = 0.5069, T_o_ = 2.0275 and f_o_ = 0.4932. From (l_o_/g)^1/2^ = 0.3162, it is deduced that Ψ(1) = 0.5069/0.3162 = 1.6030, again of the order of magnitude unity.

**Fig 5:**
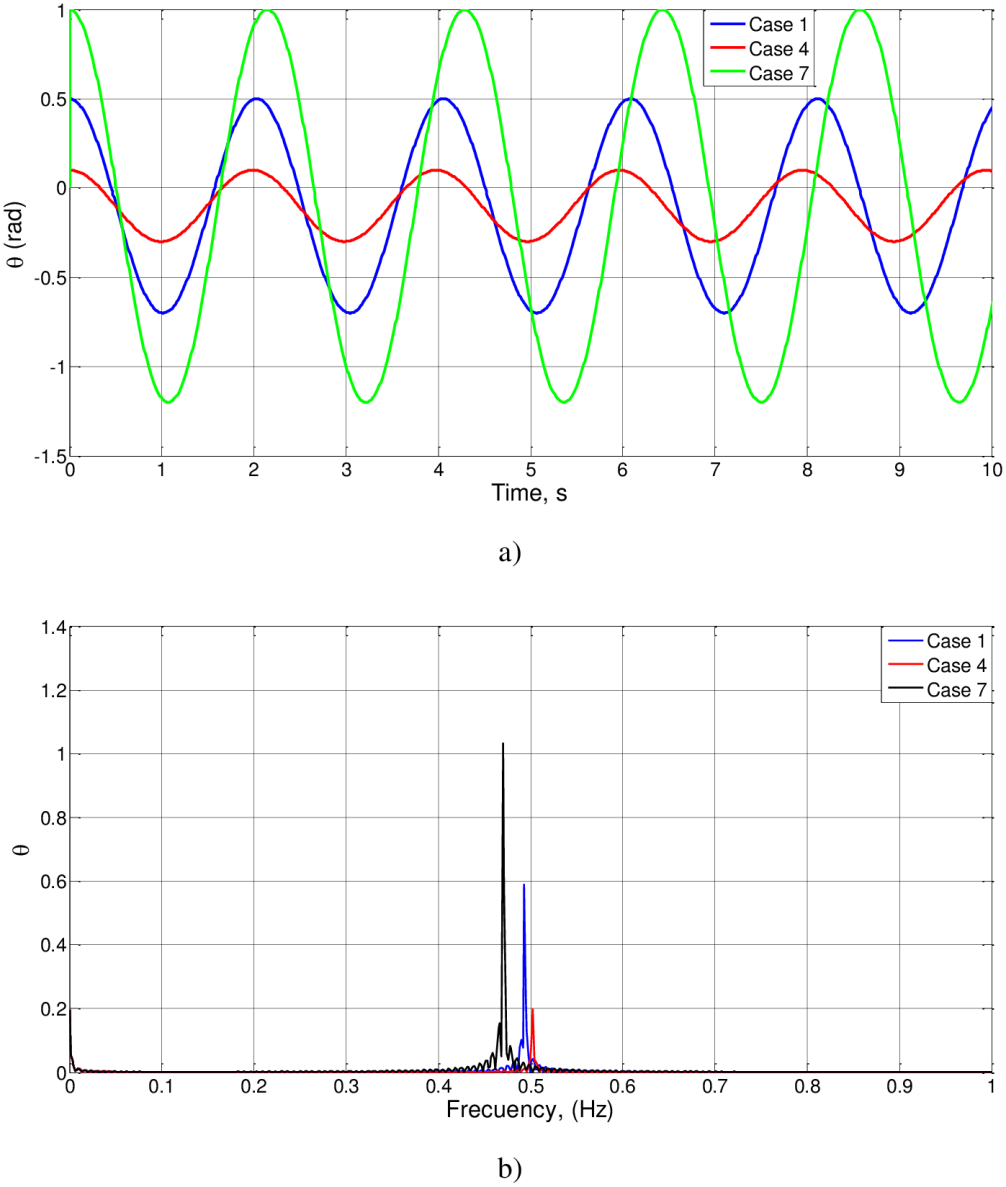
a): θ(t) cases 1, 4 and 7, b): FFT spectra for cases 1, 4 and 7.

The influence of θ is studied in cases 4, 1 and 7. The solutions of these cases are shown in Fig 5. The higher frequency corresponds to the lowest value of θ_o_: f_o_ = 0.500 (for θ_o_ = 0.1), 0.490 (for θ_o_ = 0.5) and 0.470 (for θ_o_ = 1). From these simulations, new values of the unknown function can be obtained.

Now, we check, l_o_ and g change proportionally while g and θ_o_ do so inversely (cases 4, 8 and 9). The solution of these three cases is that of case 4, which has already been presented.

**Fig 6:**
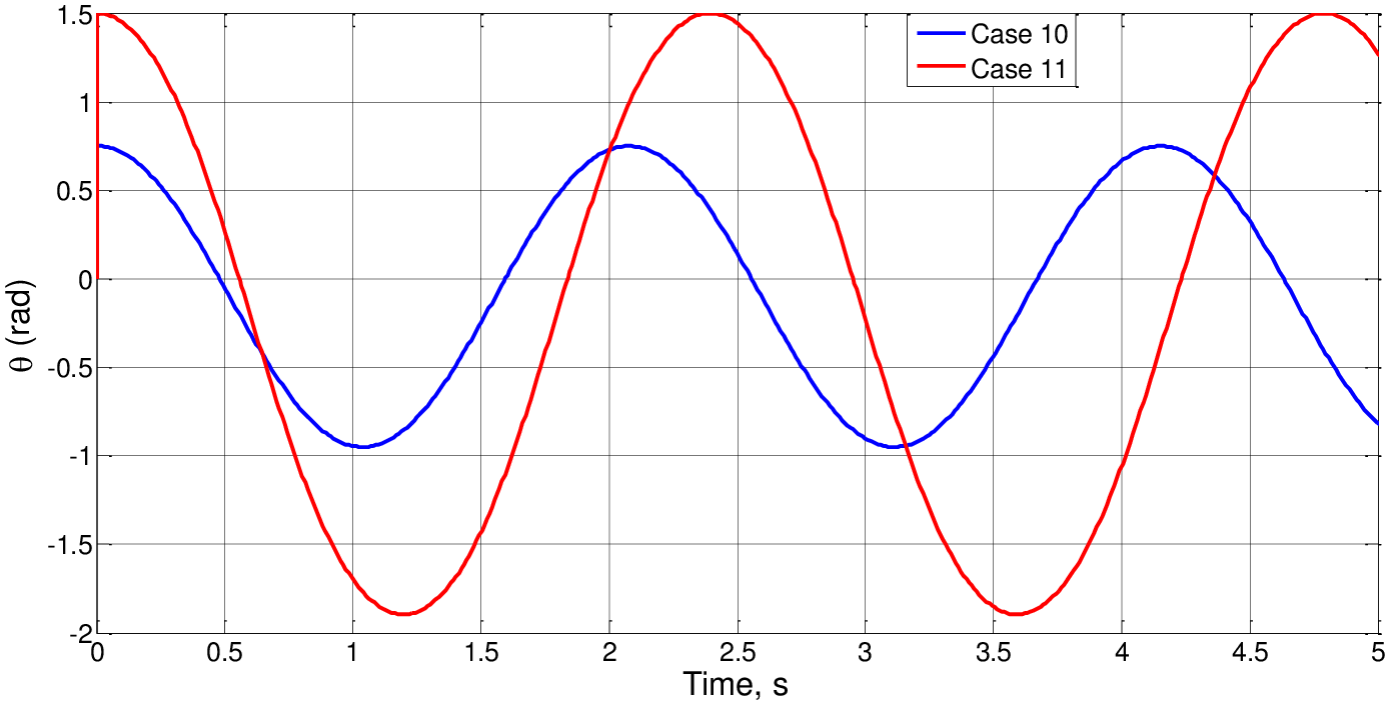
θ(t) for cases 10 and 11.

**Fig 7:**
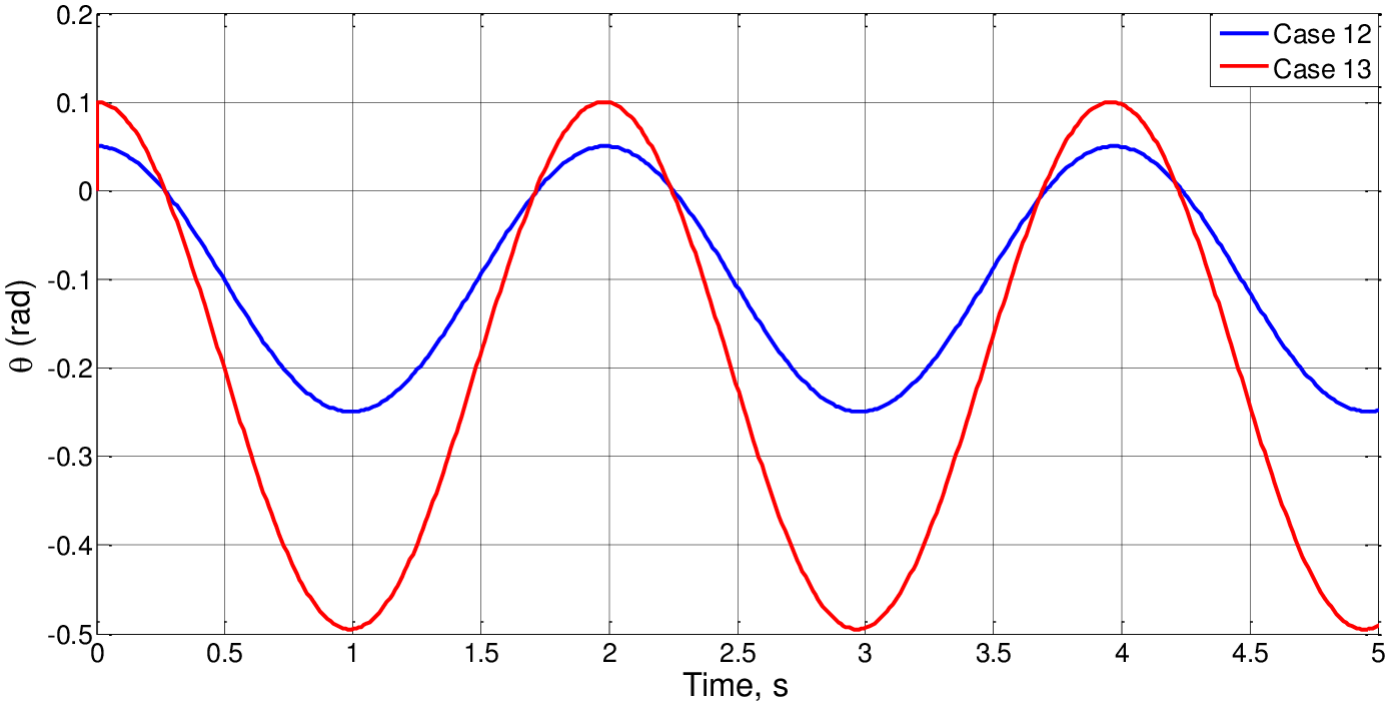
θ(t) for cases 12 and 13.

Finally, all the parameters were changed, retaining the same value for the groups l_o_/g and [a_o_/(gθ_o_)]^1/2^. For small angles (cases 10 and 11) the solution, Fig 6, is that of Eq (6) while for large angles (cases 12 and 13) the solution, Fig 7, is that of (13), all being coherent with the results of nondimensionalization.

To summarize the information provided, we can observe that Fig 3, showing the results of the numerical simulation for cases 1–3, matches the expected results of the nondimensionalization, since the dimensionless numbers are of the order of magnitude unity, which is what it is intended to demonstrate.

Likewise, Figs 4 to 7 represent the remaining cases of Table 1, in which changes are introduced into parameters of the equation of the model that vary proportionally dimensionless numbers, verifying the consistency of the results observed in these Figs.

## Application 2. Pendulum over a sliding support

**Fig 8:**
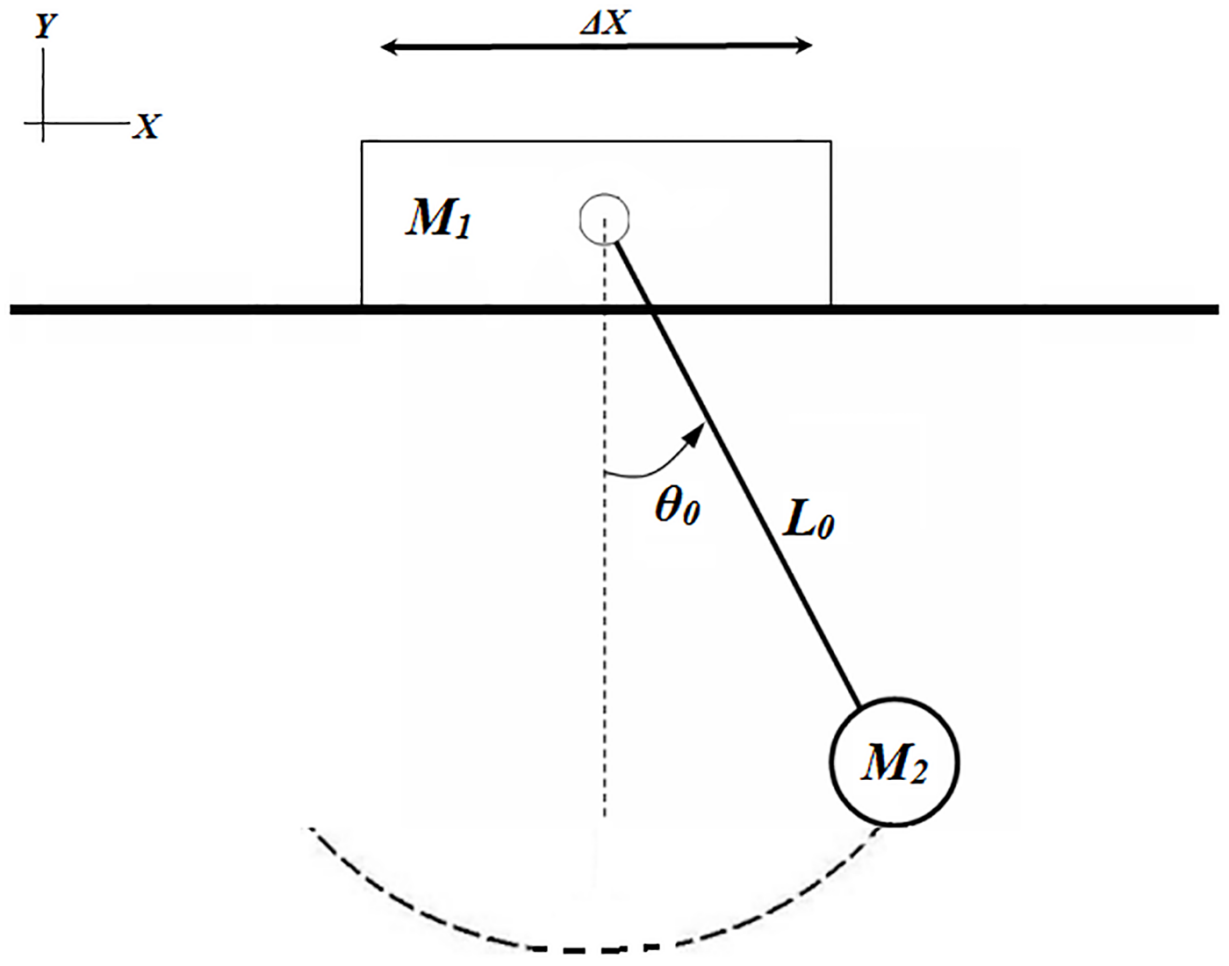
Physical scheme of application 2.

From a support of mass m_1_ that slides freely over a surface, hangs a physical pendulum of mass m_2_ and length l_o_, Fig 8. When the small ball is moved to a disequilibrium location defined by θ_o_, the ball support system oscillates freely.

To simplify the governing equations we assumed two hypotheses: θ_o_ is small and the initial velocity of the ball is zero. Under these assumptions, the mathematical model is defined by the system of coupled equations

$$\left({\text{m}}_{1}\;+\;{\text{m}}_{2}\right)\left(\frac{{\text{d}}^{2}\text{x}}{{\text{dt}}^{2}}\right)\;+\;{\text{m}}_{2}{\text{l}}_{\text{o}}\left(\frac{{\text{d}}^{2}\theta }{{\text{dt}}^{2}}\right)=0$$ (14)

$$\left({\text{m}}_{2}{\text{l}}_{\text{o}}\right)\left(\frac{{\text{d}}^{2}\text{x}}{{\text{dt}}^{2}}\right)\;+\;{\text{m}}_{2}{\text{l}}_{\text{o}}^{2}\left(\frac{{\text{d}}^{2}\theta }{{\text{dt}}^{2}}\right)\;+\;{\text{m}}_{2}{\text{gl}}_{\text{o}}\theta =0$$ (15)

with g being the gravitational acceleration. Choosing as reference quantities θ_o_, x_o_ (half of the displacement of the support in its oscillatory movement, a hidden unknown quantity) and t_o_ (half of the time required by the ball to swing from one end to other), the dimensionless variables are defined in the form:θ’ = θ/θ_o_, x’ = x/x_o_ and t’ = t/t_o_. Substituting these variables in Eqs (14) and (15) enables the dimensionless governing equations to be derived

$$\left(\frac{\left({\text{m}}_{1}\;+\;{\text{m}}_{2}\right){\text{x}}_{\text{o}}}{{\text{t}}_{\text{o}}^{2}}\right)\frac{{\text{d}}^{2}\text{x}\prime }{{\text{dt}}^{\prime 2}}\;–\;\left(\frac{{\text{m}}_{2}{\text{l}}_{\text{o}}\theta_{\text{o}}}{{\text{t}}_{\text{o}}^{2}}\right)\frac{{\text{d}}^{2}\theta \prime }{{\text{dt}}^{\prime 2}}=0$$ (16)

$$\left(\frac{{\text{m}}_{2}{\text{l}}_{\text{o}}{\text{x}}_{\text{o}}}{{\text{t}}_{\text{o}}^{2}}\right)\frac{{\text{d}}^{2}\text{x}\prime }{{\text{dt}}^{\prime 2}}\;–\;\left(\frac{{\text{m}}_{2}{\text{l}}_{\text{o}}^{2}\theta_{\text{o}}}{{\text{t}}_{\text{o}}^{2}}\right)\frac{{\text{d}}^{2}\theta \prime }{{\text{dt}}^{\prime 2}}\;+\;{\text{m}}_{2}{\text{gl}}_{\text{o}}\theta_{\text{o}}=0$$ (17)

From the first Eq (16), two coefficients emerge

$${\text{C}}_{1}=\left(\frac{\left({\text{m}}_{1}+{\text{m}}_{2}\right){\text{x}}_{\text{o}}}{{\text{t}}_{\text{o}}^{2}}\right)\;\;\text{and}\;\;{\text{C}}_{2}=\left(\frac{{\text{m}}_{2}{\text{l}}_{\text{o}}\theta_{\text{o}}}{{\text{t}}_{\text{o}}^{2}}\right)$$ (18)

giving rise to one dimensional group

$$\pi_{1,1}=\left(\frac{\left({\text{m}}_{1}+{\text{m}}_{2}\right){\text{x}}_{\text{o}}}{{\text{m}}_{2}{\text{l}}_{\text{o}}\theta_{\text{o}}}\right)$$ (19)

The order of magnitude is then given by

$${\text{x}}_{\text{o}}\;∼\;\left(\frac{{\text{m}}_{2}{\text{l}}_{\text{o}}\theta_{\text{o}}}{{\text{m}}_{1}+{\text{m}}_{2}}\right)$$ (20)

From the second Eq (17), three coefficients emerge

$${\text{C}}_{3}=\left(\frac{{\text{m}}_{2}{\text{l}}_{\text{o}}{\text{x}}_{\text{o}}}{{\text{t}}_{\text{o}}^{2}}\right),\;\;\;\;{\text{C}}_{4}=\left(\frac{{\text{m}}_{2}{\text{l}}_{\text{o}}^{2}\theta_{\text{o}}}{{\text{t}}_{\text{o}}^{2}}\right)\;\;\text{and}\;\;{\text{C}}_{5}=\left(\frac{{\text{m}}_{2}{\text{gl}}_{\text{o}}\theta_{\text{o}}}{{\text{t}}_{\text{o}}^{2}}\right)$$ (21)

which provide two dimensionless groups

$$\pi_{2,1}=\left(\frac{{\text{x}}_{\text{o}}}{{\text{l}}_{\text{o}}\theta_{\text{o}}}\right)\;\;\;\;\;\;\;\pi_{2,2}=\left(\frac{{\text{gt}}_{\text{o}}^{2}}{{\text{l}}_{\text{o}}}\right)$$ (22)

and the order of magnitude of t_o_

$${\text{t}}_{\text{o}}=\sqrt{\frac{{\text{l}}_{\text{o}}}{\text{g}}}\Psi (\pi_{1,2})=\sqrt{\frac{{\text{l}}_{\text{o}}}{\text{g}}}\Psi \left(\frac{{\text{x}}_{\text{o}}}{{\text{l}}_{\text{o}}\theta_{\text{o}}}\right)\;$$ (23)

with Ψ, an unknown function of (x_0_/l_0_θ_o_). Using the result (20), t_o_ is also given by

$${\text{t}}_{\text{o}}=\sqrt{\frac{{\text{l}}_{\text{o}}}{\text{g}}}\Psi \left(\frac{{\text{m}}_{2}}{{\text{m}}_{1}+{\text{m}}_{1}}\right)\;$$ (24)

From the relevant list of variables, m_1_, m_2_, l_o_, g, θ_o_ and the unknowns, x_o_ and t_o_, dimensional analysis provides three dimensionless groups that can be expressed as

$$\pi_{1}=\frac{{\text{m}}_{1}}{{\text{m}}_{2}}\;\;\;\;\;\;\;\;\;\pi_{2}=\left(\frac{{\text{x}}_{\text{o}}}{{\text{l}}_{\text{o}}\theta_{\text{o}}}\right)\;\;\;\;\;\;\;\pi_{3}=\left(\frac{{\text{gt}}_{\text{o}}^{2}}{{\text{l}}_{\text{o}}}\right)$$ (25)

From these groups, the solution for x_o_ and t_o_ is

$${\text{x}}_{\text{o}}={\;\text{l}}_{\text{o}}\theta_{\text{o}}\Psi_{\text{x}}\left(\frac{{\text{m}}_{1}}{{\text{m}}_{2}}\right)\;\;\text{and}\;\;{\text{t}}_{\text{o}}=\;\sqrt{\frac{{\text{l}}_{\text{o}}}{\text{g}}}\Psi_{\text{t}}\left(\frac{{\text{m}}_{1}}{{\text{m}}_{2}}\right)\;\;\;$$ (26)

Again, for t_o_, the solutions of dimensional analysis and nondimensionalization are the same, while for x_o_ the solution of dimensional analysis is less precise.

To check the results of the nondimensionalization we will simulate the cases of Table 2.

**Table 2:** Cases for the second application.

Case	1	2	3	4	5	6	7	8	9
**l**_**o**_	1	2	4	1	2	1	1	1	1
**θ**_**o**_	0.05	0.025	0.0125	0.1	0.1	0.05	0.05	0.05	0.05
**g**	10	20	40	1	2	10	10	10	10
**m**_**1**_	1	1	1	1	1	1	2	1	2
**m**_**2**_	1	1	1	1	3	2	4	3	1

**Fig 9:**
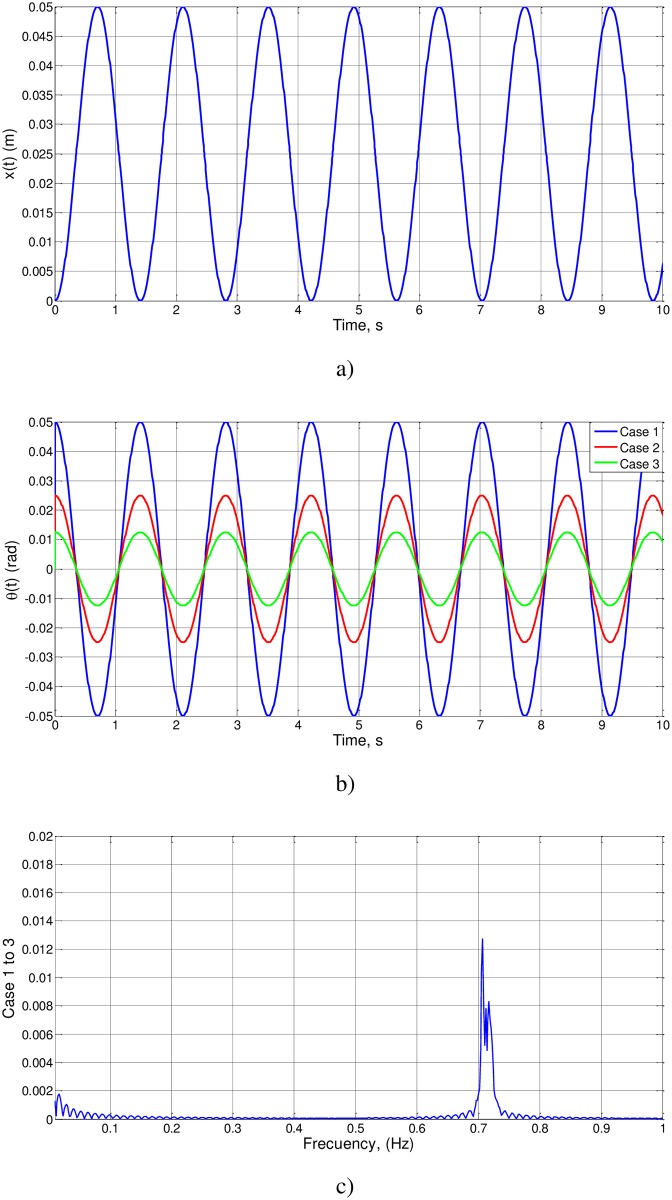
x(t), θ(t) and FFT spectra of cases 1 to 3.

Cases 1 to 3, whose numerical solution is shown in Fig 9, retain the same value for the groups l_o_/g and l_o_θ_o_, so that x_o_ and t_o_ have the same value. The solution x_o_ = 0.025 is coherent with Eq (20), which can now be written as an equality

$${\text{x}}_{\text{o}}=\left(\frac{{\text{m}}_{2}{\text{l}}_{\text{o}}\theta_{\text{o}}}{{\text{m}}_{1}+{\text{m}}_{2}}\right)$$

Also, t_o_ = 0.704s (f_o_ = 0.7101 Hz) and from (22) Ψ(0.5) = 2.2279, a value of the order of magnitude of unity, as expected.

**Fig 10:**
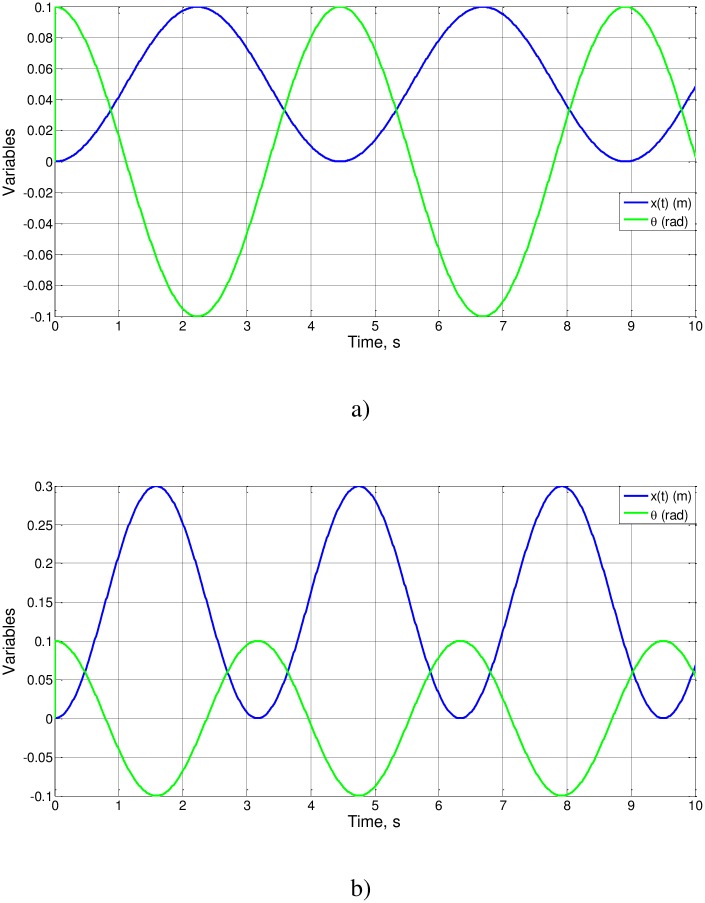
x(t) andθ(t). a) Case4 and b) case 5.

Simulations of cases 4 and 5, shown in Fig 10, provide different solutions for both unknowns, x_o_ and t_o_ according to (20) and (24): x_o_ = 0.05 m and t_o_ = 1.11 s (case 4) and x_o_ = 0.15 m and t_o_ = 0.79 s (case 5). From these values, Ψ(0.5) = 1.1107 and Ψ(0.75) = 0.79, which are of the order of magnitude of unity. Note that Ψ(1) has the same value of cases 1 to 3.

**Fig 11:**
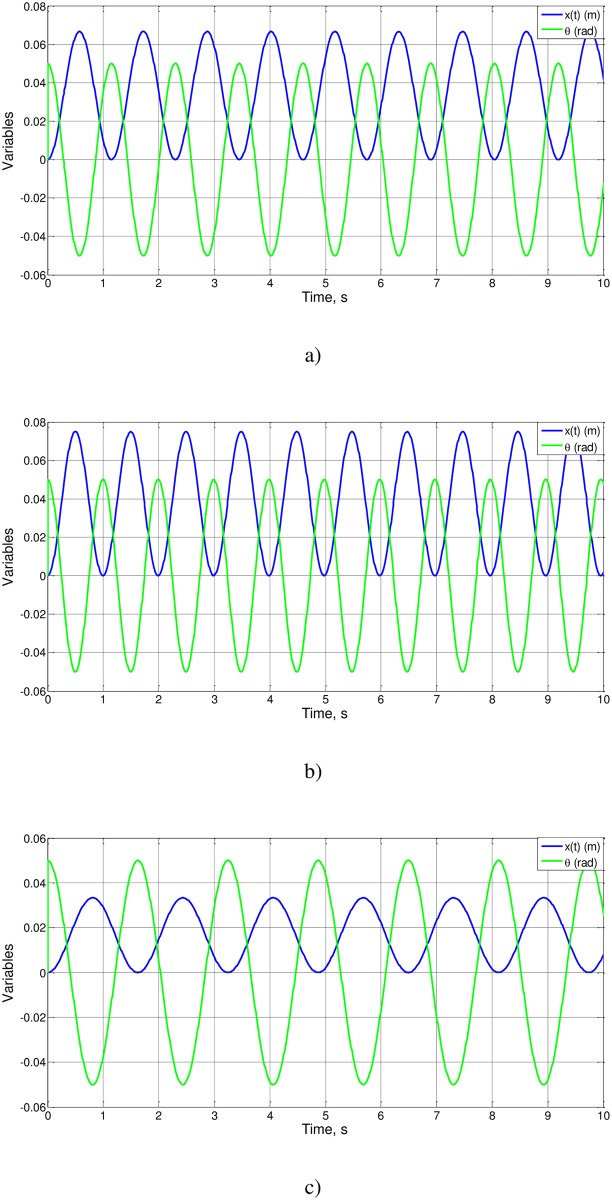
x(t) andθ(t). a) Cases 6 and 7, b) case 8 and c) case 9.

Cases 6 to 9, Fig 11, retain the values of g/l_o_ and l_o_θ_o_ while the m_2_/(m_2_+m_1_) ratio changes. Cases 6 and 7 have the same solution, while cases 8 and 9 do not, in accordance with Eqs (20) and (24). From the simulations, Ψ(0.75) = 0.79 (a solution already calculated), Ψ(0.666) = 0.91 and Ψ(0.333) = 1.28, values of the order of magnitude unity.

Here we have followed the same methodology in the previous example, showing in Fig 9 the solution of simulation for cases 1–3 of Table 2 and Figs 10 and 11, by introducing changes in the parameters that modify the dimensionless numbers in proportion. As it can be seen, in all the Figs, these changes fit perfectly with the results of the process of nondimensionalization providing values for Ψ functions of the order of magnitude unity, as it would expect.

## Application 3. Simple interaction between two species

**Fig 12:**
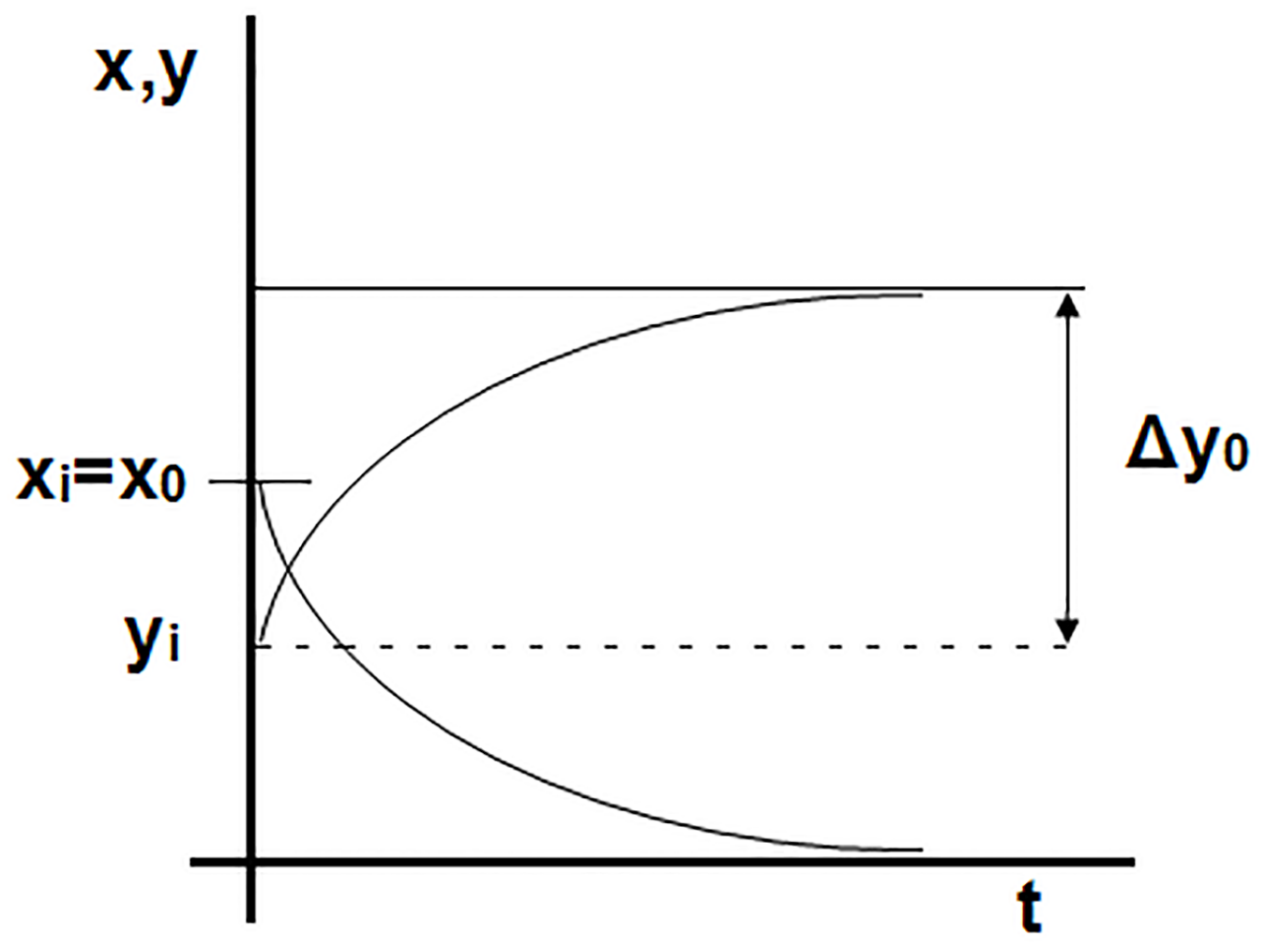
Typical solution of the simple prey-predator system.

One of the simplest models that simulates the interaction between two species is governed by the equations

$$\frac{\text{dx}}{\text{dt}}=-{\text{a}}_{\text{o}}{\text{x}(\text{y}+\text{y}}_{\text{i}})$$ (27)

$$\frac{\text{dy}}{\text{dt}}={\text{b}}_{\text{o}}{\text{x}(\text{y}+\text{y}}_{\text{i}})$$ (28)

where the coefficients a_o_ and b_o_, as well as the initial values of the variables x_i_ e y_i_, are positive, which means that the predator (variable y) increases its population asymptotically because of the decreasing of the prey population (variable x) until extinction. The general form of the solution is shown in Fig 12.

In this way, variable x takes values within the interval [x_i_,0] while variable y takes values within the interval [y_i_,y_o_-y_i_], with y_o_ the limit value of such variables. The suitable references for the nondimensionalization are then x_o_ = x_i_ for the x variable and Δy_o_ = y_o_-y_i_ for the y variable. As regards time, an unknown reference t_o_ of the order of the transient period is assumed. With this, dimensionless variables define as x’ = x/x_i_, y’ = (y-y_i_)/Δy_o_ and t’ = t/t_o_. Substituting x’, y’ and t’ in Eqs (27) and (28) yields the dimensionless governing equations of the problem. These are

$$\left(\frac{{\text{x}}_{\text{i}}}{{\text{t}}_{\text{o}}}\right)\frac{\text{dx}\prime }{\text{dt}\prime }=-{\text{a}}_{\text{o}}{\text{x}}_{\text{i}}\Delta {\text{y}}_{\text{o}}\text{x}\prime \text{y}\prime -{\text{a}}_{\text{o}}{\text{x}}_{\text{i}}{\text{y}}_{\text{i}}\text{x}\prime$$ (29)

$$\left(\frac{\Delta {\text{y}}_{\text{o}}}{{\text{t}}_{\text{o}}}\right)\frac{\text{dy}\prime }{\text{dt}\prime }={\text{b}}_{\text{o}}{\text{x}}_{\text{i}}\Delta {\text{y}}_{\text{o}}\text{x}\prime \text{y}\prime +{\;\text{b}}_{\text{o}}{\text{x}}_{\text{i}}{\text{y}}_{\text{i}}\text{x}\prime$$ (30)

The first leads to the coefficients

$$\frac{{\text{x}}_{\text{i}}}{{\text{t}}_{\text{o}}},\;\;\;\;\;\;\;\;{\text{a}}_{\text{o}}{\text{x}}_{\text{i}}\Delta {\text{y}}_{\text{o}}\;\;\;\;\;\;\;{\text{a}}_{\text{o}}{\text{x}}_{\text{i}}{\text{y}}_{\text{i}}$$

which determine two dimensionless groups

$$\pi_{1}=\left[{\text{t}}_{\text{o}}{\text{a}}_{\text{o}}{\text{y}}_{\text{i}}\right]\;\;\;\;\;\;\pi_{2}=\left[\Delta {\text{y}}_{\text{o}}/{\text{y}}_{\text{i}}\right]$$ (31)

while the second leads to the coefficients

$$\frac{\Delta {\text{y}}_{\text{o}}}{{\text{t}}_{\text{o}}},\;\;\;\;\;{\text{b}}_{\text{o}}{\text{x}}_{\text{i}}\Delta {\text{y}}_{\text{o}}\;\;\;\;{\text{b}}_{\text{o}}{\text{x}}_{\text{i}}{\text{y}}_{\text{i}}$$

which, in turn, provides the groups

$$\pi_{3}=\left[{\text{t}}_{\text{o}}{\text{b}}_{\text{o}}{\text{x}}_{\text{i}}\right]\;\;\;\;\;\;\pi_{4}=\left[\Delta {\text{y}}_{\text{o}}/{\text{y}}_{\text{i}}\right]=\pi_{2}$$ (32)

From (31) and (32), the order of magnitude of t_o_ is given by

$${\text{t}}_{0}=\left(\frac{\text{1}}{{\text{a}}_{\text{o}}{\text{y}}_{\text{i}}}\right)\Phi_{1}\left(\frac{\Delta {\text{y}}_{\text{o}}}{{\text{y}}_{\text{i}}}\right)\;\;\;\text{or}\;\;{\text{t}}_{0}=\left(\frac{\text{1}}{{\text{b}}_{\text{o}}{\text{x}}_{\text{i}}}\right)\Phi_{2}\left(\frac{\Delta {\text{y}}_{\text{o}}}{{\text{y}}_{\text{i}}}\right)$$ (33)

The ratio of this time allows us to write

$$\frac{{\text{b}}_{\text{o}}{\text{x}}_{\text{i}}}{{\text{a}}_{\text{o}}{\text{y}}_{\text{i}}}=\Phi_{3}\left(\frac{\Delta {\text{y}}_{\text{o}}}{{\text{y}}_{\text{i}}}\right)$$ (34)

As we will see later, Φ_3_(Δy_0_/y_i_) = Δy_0_/y_i_, so that the last equation allows us to relate the unknown Δy_o_ with the parameters given in the statement of the problem

$$\Delta {\text{y}}_{\text{o}}∼\left(\frac{{\text{b}}_{\text{o}}{\text{x}}_{\text{i}}}{{\text{a}}_{\text{o}}}\right)$$ (35)

and to re-write the order of magnitude of t_o_ as a function of known parameters:

$${\text{t}}_{0}=\left(\frac{\text{1}}{{\text{a}}_{\text{o}}{\text{y}}_{\text{i}}}\right)\Phi_{1}\left(\frac{{\text{b}}_{\text{o}}{\text{x}}_{\text{i}}}{{\text{a}}_{\text{o}}{\text{y}}_{\text{i}}}\right)\;\;\text{or}\;\;{\text{t}}_{0}=\left(\frac{\text{1}}{{\text{b}}_{\text{o}}{\text{x}}_{\text{i}}}\right)\Phi_{2}\left(\frac{{\text{b}}_{\text{o}}{\text{x}}_{\text{i}}}{{\text{a}}_{\text{o}}{\text{y}}_{\text{i}}}\right)$$ (36)

The application of classical dimensional analysis to this problem is immediate. Relevant list, formed by the magnitudes x_i_, y_i_, a_o_ and b_o_, lead to the dimensionless groups (π-theorem) π_1_ = x_i_/y_i_ and π_2_ = a_o_/b_o_. The solution for the dimensionless form of the unknowns Δy_o_/y_i_ and t_o_, a_o_, x_i_ (or t_o_, b_o_, y_i_) are then

$$\Delta {\text{y}}_{\text{o}}={\text{y}}_{\text{i}}\Psi_{1}\left(\frac{{\text{x}}_{\text{i}}}{{\text{y}}_{\text{i}}},\;\frac{{\text{a}}_{\text{o}}}{{\text{b}}_{\text{o}}}\right)\;\;\text{and}\;\;{\text{t}}_{0}=\frac{1}{{\text{a}}_{\text{o}}{\text{x}}_{\text{i}}}\Psi_{2}\left(\frac{{\text{x}}_{\text{i}}}{{\text{y}}_{\text{i}}},\;\frac{{\text{a}}_{\text{o}}}{{\text{b}}_{\text{o}}}\right)$$ (37)

a less precise solution of that provided by nondimensionalization.

Now some cases are simulated to check the above results, Table 3.

**Table 3:** Cases of the prey-predator system.

Case	1	2	3	4	5	6	7
**a**_**o**_	1	2	1	1	2	2	1
**b**_**o**_	1	4	2	3	1	3	3
**x**_**i**_	1	3	1	1	2	2	4
**y**_**i**_	1	1.5	2	3	1	3	2

**Fig 13:**
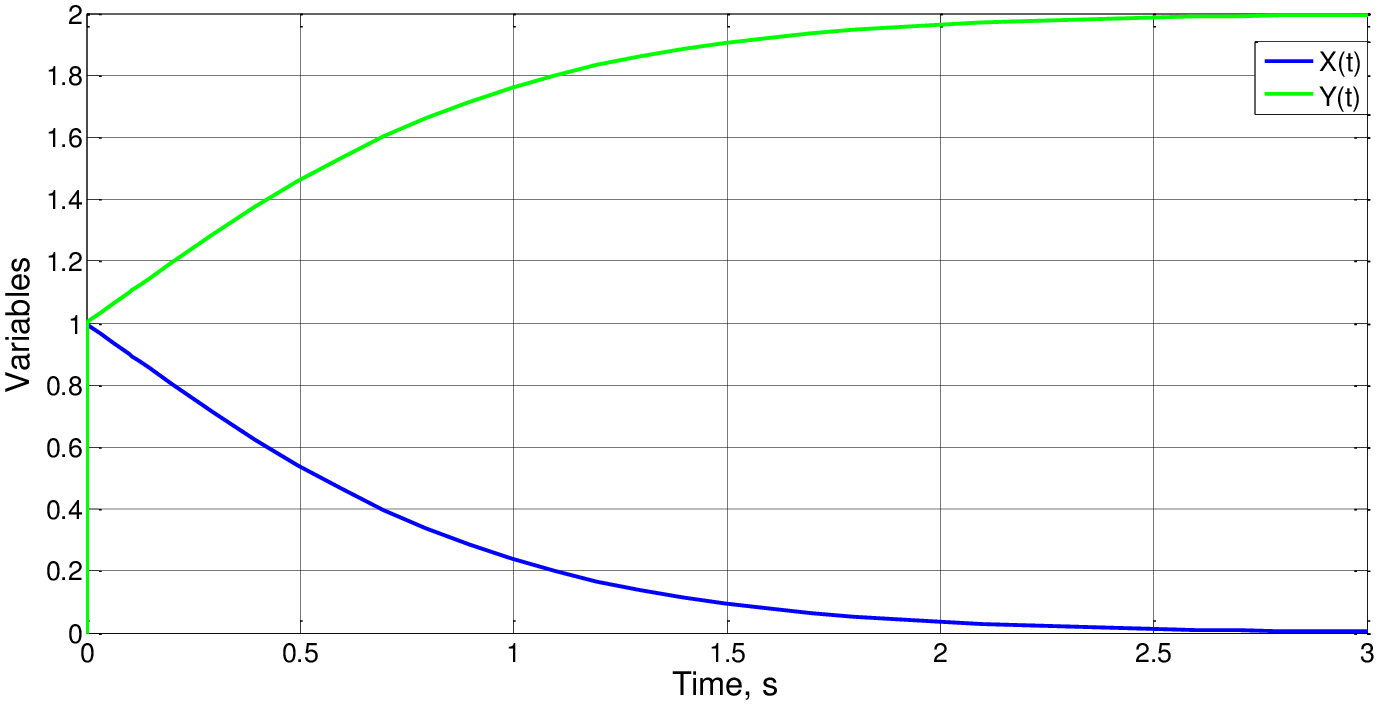
x(t) and y(t) for case 1.

Case 1 is simulated in Fig 13. Since the curves are asymptotic, we choose t_o_ as the time required by x variable to reduce its value to 0.2x_i_. From the numerical solution, t_o_ = 1.099 and Δy_o_ = 1. Using the expressions (33) and (34), the values (of order of magnitude unity as expected) of the functions ϕ_1_, ϕ_2_ and ϕ_3_, for argument unity, are obtained:

$$\Phi_{3}(1)=1,\;\Phi_{1}(1)=\Phi_{2}(1)=1,099$$

**Fig 14:**
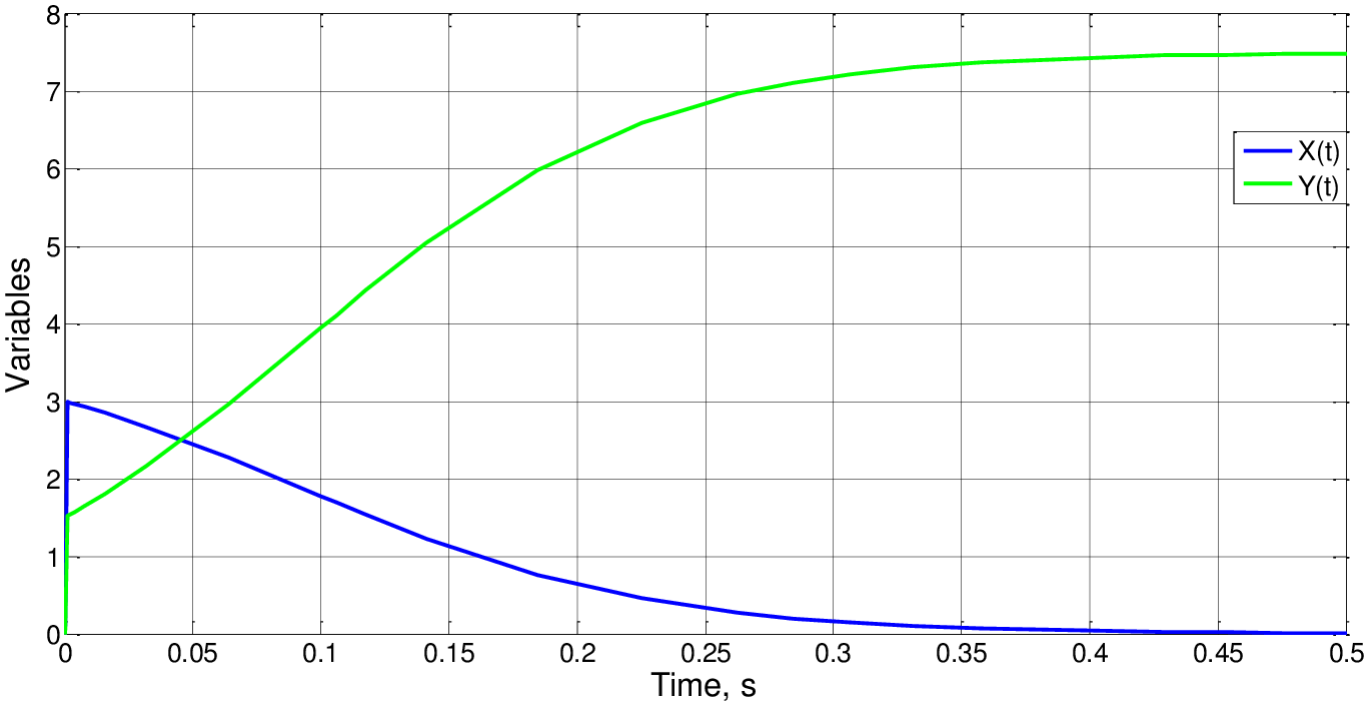
x(t) and y(t) for case 2.

Taking case 2, whose simulation shown in Fig 14, we obtain Δy_o_ = 6, so that Ψ_3_(4) = 4. Generalizing this result, i.e., making Ψ_3_(c) = c, the expression of Δy_o_, in terms of the parameters of the statement (35), is demonstrated.

**Fig 15:**
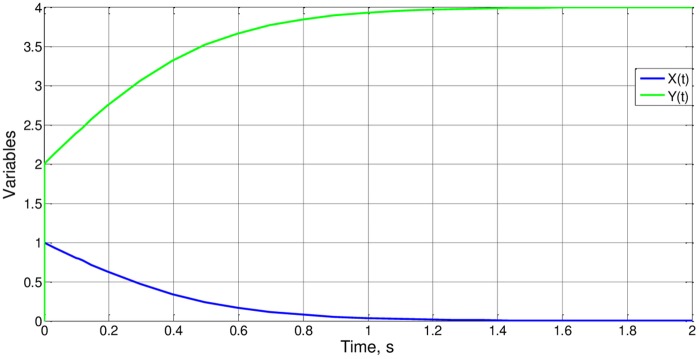
x(t) and y(t) for cases 3 to 6.

**Fig 16:**
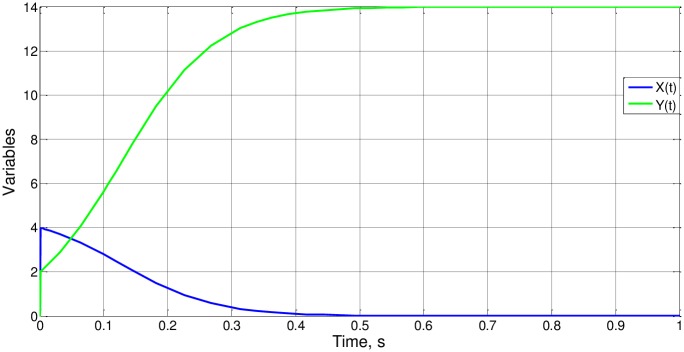
x(t) and y(t) for case 7.

To finish, we describe some cases in which all the parameters presented vary. Cases 3 to 6 retain the same value for the group b_o_x_i_/a_o_y_i_ so that Ψ_1_ (and Ψ_2_) do not change. Since b_o_x_i_/a_o_y_y_ = 1 for all cases, Ψ_1_(1) = Ψ_2_(1) = 1,099according to the results of case 1. In this way, from (35) t_o_ = 1.099a_o_y_i_ = 1.099b_o_x_i_, an expression that is coherent with the simulation, Fig 15. As for case 7, Fig 16, b_o_x_i_/a_o_y_y_ = 6, and t_o_ = 0.24, from simulation. It is deduced that Ψ_2_(6) = 0.96, a value of the order of magnitude unity as expected.

As we can see from these results, with normalized nondimensionalization technique, we extract dimensionless numbers of the order of magnitude unity that leads to solutions which fits perfectly with the obtained results of the numerical simulation, where we can check from the Figs that the acquired values of variables of interest, provide values for the functions Ψ, also of the order of magnitude unit, in each case.

## Final comments and conclusions

Although many authors consider that dimensional analysis and nondimensionalization are two similar approaches in the search for the dimensionless groups that rule a given problem, both formulated by ordinary or partial differential equations, coupled or not, the application of the second approach may lead to a more precise solution under certain hypotheses. The choice of suitable references, for which a profound knowledge of the physical phenomenon involved is required, that allow dimensionless variables to be defined by a range of values within the interval [0–1], provides the same order of magnitude to the coefficients of the new ‘normalized’ equations. The complete set of independent ratios between these coefficients has two inherent properties: they can be physically interpreted in terms of a balance of the quantities that counteract in the problem, and they are of the order of magnitude unity.

The explanation of the advantage of the nondimensionalization may seem over-subtle but it is not. Classical dimensional analysis starts with the definition of the relevant list of variables—parameters and quantities—that influence the problem, including the unknowns being sought. However, no requirements are imposed on these variables except a physical reasoning, frequently insufficient, that justifies their inclusion in that list.

This, together with a definition of the dimensional basis (for example, length, mass and time in mechanics), allows us to obtain the dimensionless groups, to which, on the one hand, it is difficult to attribute a balance and, on the other hand, it is impossible to attribute an order of magnitude. By contrast, the choice of references to make the variables dimensionless in the proposed nondimensionalization processes is not arbitrary, but is subject to a kind of discrimination that normalizes the dimensionless equations that these variables define. The difference between dimensional analysis and nondimensionalization, as proposed in this work, means that many of the direct groups that emerge in dimensional analysis, such as mass ratios or geometrical factor, do not appear with nondimensionalization which leads to a more precise solution, as the above applications illustrate.

Concerning what is referred to the future prospects of the use of this technique, we can say that during tens of years, the process of nondimensionalization has been applied (generally to models defined by coupled partial differential equations) in a manner that we might call classical, and the scientific community has agreed with the existence of dimensionless numbers with orders of magnitude far greater than the unity. Can they have any physical meaning, in terms of balance, these numbers? Of course not, but there are texts in which recognizes them this meaning. They are poorly learned numbers and now this is difficult to renew. However, the processes made by ordinary differential equations are not usually subject of controversy in the scientific literature. It should be noted that the problems dealt with in this work refer to physical processes governed by natural laws. is an issue to which we will dedicate efforts in the future. We can say, without fear of error that the field is open, especially in coupled problems, and we hope to be able to spread our humble contributions to the scientific community.
